# Proposal of a “Checklist” for endodontic treatment

**DOI:** 10.4317/jced.51126

**Published:** 2014-04-01

**Authors:** Víctor Díaz-Flores–García, Bernardo Perea-Pérez, Elena Labajo-González, Andrés Santiago-Sáez, Rafael Cisneros-Cabello

**Affiliations:** 1 Collaborator of the Spanish Observatory for Dental Patient Safety (OESPO). Professor of the School of Health Sciences. Universidad Europea de Madrid; 2 Director of the Spanish Observatory for Dental Patient Safety (OESPO). Director of the School of Legal and Forensic Medicine of Madrid. School of Medicine. Universidad Complutense de Madrid; 3 Secretary of the Spanish Observatory for Dental Patient Safety (OESPO). Professor of the School of Legal and Forensic Medicine of Madrid. School of Medicine. Universidad Complutense de Madrid; 4 Member of the Spanish Observatory for Dental Patient Safety (OESPO). Professor of the School of Legal and Forensic Medicine of Madrid. School of Medicine. Universidad Complutense de Madrid

## Abstract

Objectives: On the basis of the “Surgical Checklist” proposed by the WHO, we propose a new Checklist model adapted to the procedures of endodontic treatment. Study Design: The proposed document contains 21 items which are broken down into two groups: those which must be verified before beginning the treatment, and those which must be verified after completing it, but before the patient leaves the dentist’s office. Results: The Checklist is an easy-to-use tool that requires little time but provides, order, logic and systematization by taking into account certain basic concepts to increase patient safety. Discussion: We believe that the result is a Checklist that is easy to complete and which ensure the fulfillment of the key points on patient safety in the field of endodontics.

** Key words:**Checklist, endodontics, patient safety, adverse event.

## Introduction

In the early nineties, a work by Leape *et al.* (1) made it clear that, of the adverse events which were reported to have occurred in health care, two-thirds were preventable. Although the damage which could be caused by health care practice was always known, it was not until then that awareness of the problem’s real importance came into existence and its quantification began.

Another fundamental study in terms of increasing awareness about this problem was one completed in 1999 by the Institute of Medicine of the National Academics (IOM) (2), which pointed out that 44,000 to 98,000 unnecessary deaths took place in the United States as a result of health care itself.

These data logically caused concern amongst professionals, as well as political organizations and health care entities, and they led to a promotion of the studies and measures for improvements in patient safety over the last ten years (3,4).

In light of all these data, the World Health Organization created the “World Alliance for Patient Safety,” which established the basic guidelines for patient protection and in 2007 set as one of its main objectives the highest level “Global Patient Safety Challenge: Safe Surgery Saves Lives,” having published the “Surgical Safety Checklist” in 2008 ( Table 1). This Surgical Checklist was then proposed as a simple, easy-to-use tool that would ensure the fulfillment of key patient safety elements. The checklist’s development was based on three principles: simplicity, widespread applicability and accountability (5).

**Table 1:**
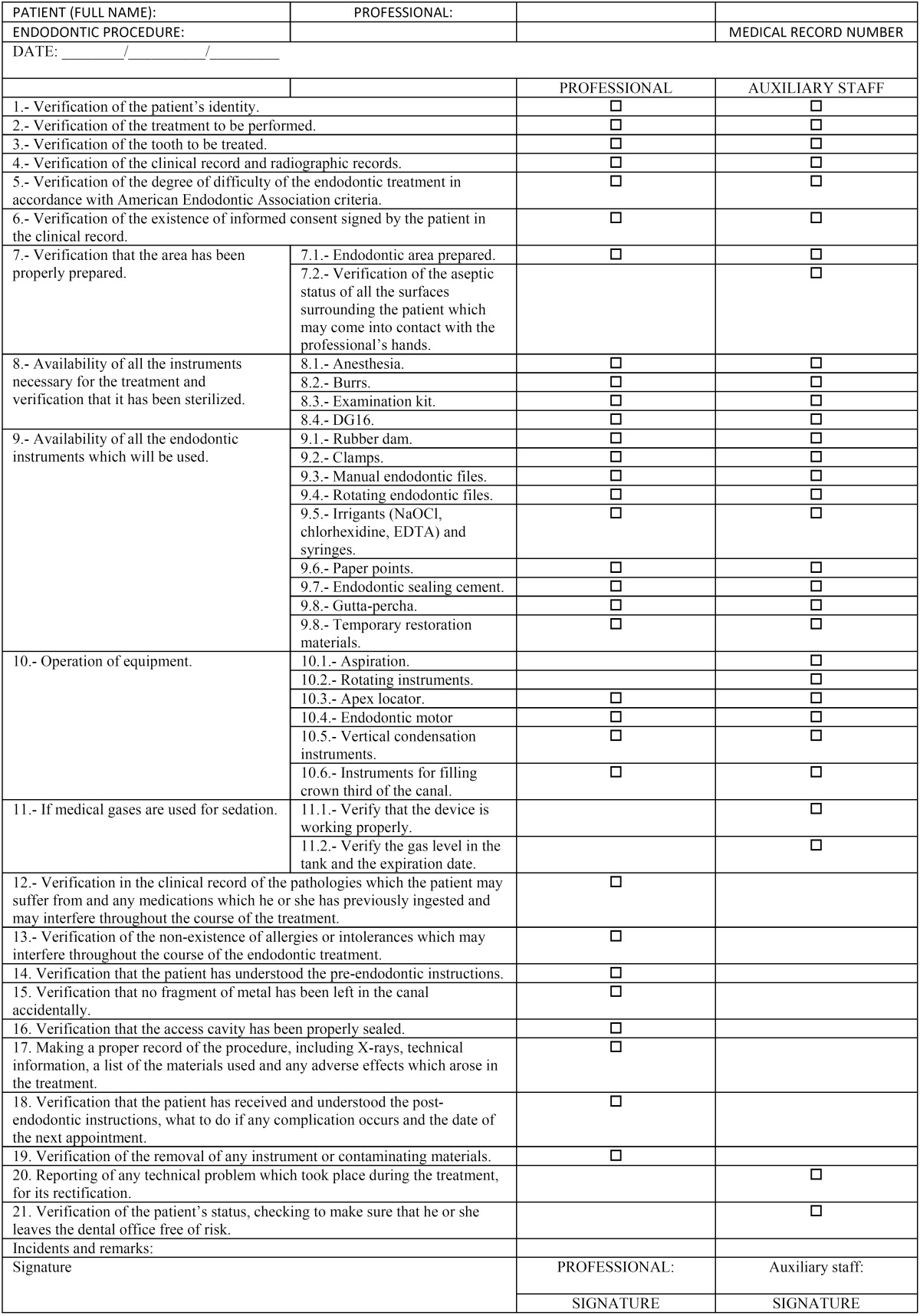
Surgical Safety Checklist.

The effective nature of this checklist for verification on the increase in surgical safety for patients has been demonstrated on a widespread basis in various studies (6-12).

In the same way that the document which presents the Surgical Checklist places an emphasis on the need for adaptation to diverse circumstances, habits and specializations in surgical activity, we believe that the principles formulated by the “World Alliance for Patient Safety” for completing and applying the Checklist may also be applied to dental treatments (13). It is from this perspective that we deal with its adaptation to the field of endodontics.

Proposing a Checklist or Verification List which meets the conditions of the “World Alliance for Patient Safety” specifically adapted to the field of endodontics is the objective of this work.

## Material and Methods

Because the Surgical Checklist of the “World Alliance for Patient Safety” is intended mainly for major hospital surgery procedures, the particularities which differentiate the procedures in dental treatments from those procedures are obvious.

Some of these peculiarities are as follows:

• Their ambulatory nature. This leads to a lack of control over potential complications which occur after treatment.

• The majority use of local or locoregional anesthesia, occasionally with the support of sedation procedures.

• The completion of these procedures at many small centers, which are generally private. This dispersion makes it difficult to collect data on adverse events and spread a culture of patient safety.

• The limited number of health care staff members who usually take part in this type of interventions, and therefore the existence of an outside observer is infrequent.

• The limited scope of most of the procedures, which are not as invasive as in surgical activity, and especially in the surgical activities in hospitals.

## Results

- Proposed Document (Appendix A)

The document contains 21 items which must be verified, some by the auxiliary staff in charge of the Checklist, and others by the health care professional, as well as a third set by both.

The 21 items are divided into two sets: those which must be verified before beginning the endodontic treatment and those which must be verified after it is completed, but always before the patient leaves the dental office.

• Items to be verified before beginning the endodontic treatment:

1. Verification of the patient’s identity (by the dentist and by the auxiliary staff).

2. Verification of the treatment to be performed (by the dentist and by the auxiliary staff).

3. Verification of the tooth to be treated (by the dentist and by the auxiliary staff).

4. Verification of the clinical record and the radiographic records (by the dentist and by the auxiliary staff).

5. Verification of the level of difficulty of the endodontic treatment in accordance with the criteria of the Ameri-can Endodontic Association (by the dentist).

6. Verification of the existence of the informed consent completed by the patient in the clinical record (by the dentist and by the auxiliary staff).

7. Verification that the area to be treated has been properly prepared:

7.1. Endodontic area prepared (by the dentist and by the auxiliary staff).

7.2. Verification of the aseptic status of all the surfaces surrounding the patient, which may come into contact with the professional’s hands (by auxiliary staff).

8. Availability of all the instruments necessary for the treatment, and verification that it has been sterilized.

8.1. Anesthesia (by the dentist and by the auxiliary staff).

8.2. Burrs (by the dentist and by the auxiliary staff).

8.3. Examination kit (by the dentist and by the auxiliary staff).

8.4. DG16 probe (by the dentist and by the auxiliary staff).

9. Availability of all the endodontic instruments which will be used.

9.1. Rubber dams (by the dentist and by the auxiliary staff).

9.2. Clamps (by the dentist and by the auxiliary staff).

9.3. Manual endodontic files (by the dentist and by the auxiliary staff).

9.4. Rotating endodontic files –if they are necessary– (by the dentist and by the auxiliary staff).

9.5. Irrigants (NaOCl, chlorhexidine, EDTA) and syringes (by the dentist and by the auxiliary staff).

9.6. Paper points (by the dentist and by auxiliary staff).

9.7. Endodontic cement (by the dentist and by the auxiliary staff).

9.8. Gutta-percha (by the dentist and by the auxiliary staff).

9.9. Temporary restoration materials (by the dentist and by the auxiliary staff).

10. Operation of equipment.

10.1. Aspiration (by the auxiliary staff)

10.2. Rotating instruments (by the dentist and by the auxiliary staff).

10.3. Apex locator (by the dentist and by the auxiliary staff).

10.4. Endodontic motor –in the event of using rotating files– (by the dentist and by the auxiliary staff).

10.5. Vertical condensation instruments –if this technique is used– (by the dentist and by the auxiliary staff).

10.6. Instruments for filling the crown third of the canal –if this technique is used– (by the dentist and by the auxiliary staff).

11. If medical gases are used:

11.1. Verify that the device is working properly (by the auxiliary staff).

11.2. Verify the level of gas in the tank and the expiration date (by the auxiliary staff).

12. Verification of the clinical record of pathologies which the patient may suffer from and the medicines which the patient has ingested beforehand that might interfere throughout the course of the treatment (by the dentist).

13. Verification of the nonexistence of allergies or intolerances which may interfere throughout the course of the endodontic treatment (by the dentist).

14. Verification that the patient has understood the pre-endodontic instructions (by the dentist).

• Items to be verified immediately after performing the endodontic treatment (before the patient leaves the site where the endodontic treatment took place):

15. Verification that no metal fragments have been left in the canal accidentally (by the dentist).

16. Verification that the access cavity is properly sealed (by the dentist).

17. Recording the procedure, including X-rays, technical data, a list of the materials used and any adverse effects produced in the treatment (by the dentist).

18. Verification that the patient has received and understood the post-endodontic instructions, what to do if a complication arises and the date of the next appointment (by the dentist).

19. Verification of the removal of any instrument or contaminating material (by the auxiliary staff).

20. Reporting of any technical problem that arose during the treatment for its rectification (by the auxiliary staff).

21. Verification of the patient’s status, verifying that the patient leaves the site risk-free (by the dentist).

- Procedure

The Checklist for endodontic treatment was designed for completion by one single person, who should be a member of the auxiliary staff with sufficient training on patient safety. This person shall fill out the sections corresponding to the auxiliary staff and shall ask the professional, whether a dentist or endodontist, about the sections for which he or she is responsible. They must have the ability to regulate and even interrupt the procedure if any of the verifications is not properly completed. Each item on the list must be verified before continuing on with the following step of the treatment.

Completion of the Checklist shall take place at two times:

• Before beginning the procedure, the items which correspond to the auxiliary staff prior to the treatment, and immediately before anesthetization and beginning the procedure, shall be verified; the professional shall be asked about the aspects prior to the treatment for which he or she is responsible.

• Upon completion of the endodontic procedure, but before the patient leaves the dental office, the person responsible for completing the Checklist shall ask the professional about the post-treatment aspects for which the professional is responsible.

Last of all, after verifying the proper completion of all the information, the person responsible for the Checklist shall sign the document. Afterwards, this person must give the Checklist to the professional, who shall also verify it in its entirety before signing it. The document will be included in the patient’s clinical record.

As for the items prior to the endodontic treatment, at the beginning of the Checklist, they are habitual and com-mon to any dental treatment: identifying the patient, confirming the procedure to be performed and the tooth where the treatment will be carried out. This basic verification shall be performed by both the professional and by the auxiliary staff. The auxiliary staff must verify the availability of the clinical record and the radiographic records necessary to perform the treatment. The auxiliary staff shall also verify the existence of an properly signed informed consent document if this is necessary (Act 41/2002). These two aspects must also be verified by the professional, who must also make certain that the patient has understood the procedure which is to be performed, for what reason or purpose it is performed, and what potential complications may arise throughout the procedure. Likewise, the existence of prior pathologies or medications taken prior to the treatment which might affect it must be verified.

Both the auxiliary staff and the professional must verify all of the instruments and materials necessary for the treatment, including all of the endodontic materials which may be used. If sedative gases are used, the auxiliary staff must verify that the equipment is working properly and contains the proper level of gases.

As for the items after the end of the endodontic treatment, most must be verified while the patient still remains at the dental office: verification that no materials or instruments have been left in the treated area, and that the relevant information on the procedure (and on any possible complications) has been stated in the clinical record.

Last of all, before the patient leaves the dental office, it must be verified that he or she is clinically well, that he or she has understood the post-endodontic instructions, and that he or she knows what to do if any complications arise. It must also be verified that the patient leaves the dental office with proper accompaniment, if necessary.

The auxiliary staff must report any technical problem that has arise, as well as removing all materials which are at risk of biological contamination.

At the end of the entire process, the document must be signed by both the professional and the member of the auxiliary staff responsible for completing it. Both must verify, before signing, that the Checklist has been fully completed. The Checklist must be attached to the clinical record.

## Discussion

A verification list or checklist is an easy and quick tool to use for verifying the elements involved in patient safety and avoiding errors or distractions.

It does not include concepts which a clinic specializing in endodontic treatment has not performed before, but it provides order, logic and systematization to the treatment and thereby increases patient safety.

Its simplicity means that it requires little time from the clinical team.

The fact that it is included in the patient’s clinical record may be of significant value if any legal claims occur.

Due to all of this, we believe that this checklist for endodontic treatment is clearly a positive tool for the dental team.

The purpose of this Checklist for Endodontic Treatment (Appendix A) is to serve as a model adapted for the practice of ambulatory endodontic treatment. Because of this, contrary to what is proposed by the World Alliance for Patient Safety, it is completed at two times (instead of three) and only requires the intervention of one person holding responsibility between the auxiliary staff and the dentist who will be performing the treatment.

Endodontic treatment is one of the most common procedures in everyday clinical practice, and it is one of those which produces the most problems related with patient safety: adverse events caused by the use of sodium hypochlorite, allergy to latex, the breakage of endodontic files and burrs, the accidental ingestion and aspiration of foreign bodies, etc. (14-17).

Although there may be different ways of performing a proper endodontic treatment, the adverse events which may take place are generally the same, and therefore it is very important to pay special attention to patient safety. A Checklist may prevent such events.

Five years after the publication of the book “To Err is Human,” in which the IOM made clear in 1999 the numerous unnecessary deaths caused by health care itself in the United States, in 2005 Lucian et al. published a review in which they pointed out the efforts made to enhance patient safety both in the United States and around the world (1-2).

In 2006, Espin *et al.* carried out a study evaluating the persistence of unsafe clinical and surgical practices in everyday health care, highlighting the need to promote a culture of patient safety (6).

In 2012, Weiser et al. published a work on the application and validation of a Surgical Checklist in emergency surgical interventions (7). Similarly, Peyré et al. published a study in 2010 on the use of a Surgical Checklist in laparoscopic surgery interventions (9).

The reports by Wilson *et al.* (2010), Keane *et al.* (2010) , Clark *et al.* (2010) and Vats *et al.* (2010)highlight the application of the “Surgical Safety Checklist” of the “World Alliance for Patient Safety” (WHO) in different fields within medical-surgical practice (8,10-11).

Due to the efforts made, this culture of Patient Safety has gradually been spread to include odontostomatological practice (18). Accordingly, in 2011 Perea et al. published a proposed Checklist for ambulatory oral surgery. There are no checklists in other fields of dental practice, despite the fact that their effectiveness has been demonstrated and validated in many different fields of health care (6-12).

## Conclusions

We believe that the proposed Checklist for Endodontic Treatment is a tool which is easy to use and which ensures the fulfillment of the key elements of patient safety in the field of endodontics.

This type of checklists also offers the possibility of including data on adverse events, recording them and repor-ting them, which leads to an improvement in dental safety for patients, especially because there is no existing bibliography involving these cases in the dental literature.
